# The microbiological features and risk factors for liver abscess after thermal ablation of liver cancer

**DOI:** 10.3389/fcimb.2025.1567105

**Published:** 2025-06-06

**Authors:** Xiurong Ding, Yanhua Yu, Ming Chen, Yanfang Kang, Jinli Lou

**Affiliations:** Department of Clinical Laboratory, Beijing Youan Hospital, Capital Medical University, Beijing, China

**Keywords:** liver cancer, thermal ablation, liver abscess, risk factor, microbiological features

## Abstract

**Objectives:**

Liver abscess is a severe complication that can occur following thermal ablation of liver cancer, with an incidence rate ranging from 0.2% to 2.4%. This study aimed to identify risk factors associated with the development of post-ablation liver abscesses and to characterize the microbiological features of the isolated pathogens.

**Methods:**

A matched case-control study was conducted at Beijing Youan Hospital from January 2018 to December 2023. Cases were defined as patients who developed liver abscesses within three months following ablation therapy, while controls comprised patients who did not develop such abscesses. Clinical and microbiological data were collected and analyzed. The relevant independent risk factors for the occurrence of liver abscesses were identified and assessed using multivariate logistic regression analysis.

**Results:**

Post-ablation liver abscesses predominantly occurred in male patients aged 60 years or older, typically manifesting around six days after the procedure. Common symptoms included fever, chills, abdominal distension, and abdominal pain. Multivariate analysis identified diabetes mellitus (OR=3.215; 95% CI 1.330–7.771), history of abdominal surgery (OR=2.810; 95% CI 1.074–7.353), biliary disease (OR=18.832; 95% CI 2.291–154.795), and elevated ALP levels (OR=1.010; 95% CI 1.002–1.019) as independent risk factors for post-ablation liver abscesses. Among the 61 patients with liver abscesses, a total of 78 bacterial strains were isolated from the abscess fluid, with Gram-negative bacteria accounting for 75.6% of the isolates. *Escherichia coli* (30.8%) and *Klebsiella pneumoniae* (20.5%) were the most frequently identified pathogens. Drug sensitivity testing revealed that both *Escherichia coli* and *Klebsiella pneumoniae* exhibited high susceptibility to Amikacin, Cefoxitin, Ceftazidime, Imipenem, Meropenem, and Piperacillin-tazobactam.

**Conclusions:**

Post-ablation liver abscesses are primarily caused by Gram-negative bacteria. A history of diabetes, prior abdominal surgery, biliary disease, and elevated ALP levels are significant risk factors influencing the development of post-ablation liver abscesses.

## 1 Introduction

Liver cancer is one of the most prevalent digestive system malignancies and ranks second as a cause of cancer-related mortality worldwide (Bray et al., 2024). For both primary and metastatic liver cancers, especially when surgical resection is not an option, thermal ablation has become a widely recognized primary therapeutic approach (Xie et al., 2023). Radiofrequency ablation (RFA) and microwave ablation (MWA) are the predominant modalities for local thermal ablation, achieving cure rates comparable to surgery in early-stage patients (Dong et al., 2014; Fu et al., 2014). Even for larger tumors, favorable outcomes can be achieved through meticulous procedural planning (Groeschl et al., 2014; Yang et al., 2023). Despite its relative safety and minimally invasive nature, thermal ablation can lead to severe complications such as intra-abdominal hemorrhage, hepatic abscess, biliary fistula, intestinal perforation, and tumor seeding, among others (Liang et al., 2009; Spiliotis et al., 2021). Hepatic abscess is one of the most common and serious post-ablation complications, with an incidence ranging from 0.2% to 2.4%, which can prolong hospital stays and significantly impact patient mortality (Shibata et al., 2003; Park et al., 2017).

Understanding the relevant risk factors for the development of liver abscess following ablation surgery is key to reducing this complication. However, current literature focuses more on clinical management and treatment strategies than on systematic risk factors analysis (Liang et al., 2009; Ding et al., 2013), and small sample sizes limit generalizability (Kwak et al., 2023). Timely and effective antimicrobial therapy not only significantly reduces mortality but also effectively decreases the length of hospital stay for patients with liver abscess. Given regional variations in pathogens, identifying local pathogen spectra and antibiotic resistance patterns is critical for effective treatment. The aim of this study was to retrospectively analyze the microbiological features of post-ablation hepatic abscesses and identify the risk factors contributing to their development.

## 2 Materials and methods

### 2.1 Study subjects

This retrospective study analyzed 61 patients who developed post-ablation liver abscesses at Beijing Youan Hospital (Beijing Hepatitis Research Center) between January 2018 and December 2023. The diagnosis of post-ablation liver abscesses was confirmed based on the following criteria: (1)Clinical manifestations: a persistent fever exceeding 38.5°C for more than three consecutive days; a peripheral blood leukocyte count greater than 10×10^9/L or less than 4×10^9/L; recurrent episodes of chills and fever, or the development of septic shock. (2) specific imaging findings indicative of abscess formation, (3) positive bacterial cultures from pus samples, (4) evidence of percutaneous drainage or surgical intervention, and (5) onset occurring within three months following thermal ablation.

Exclusion Criteria:Patients with liver abscesses that developed more than three months post-ablation; those who developed abscesses following transarterial chemoembolization (TACE), and those with a history of prior liver abscesses were excluded.

For the control group, 3521 patients who underwent thermal ablation at our hospital between 2018 and 2023 without developing liver abscesses were initially identified. Using propensity score matching (PSM) based on age and sex, 61 matched patients were selected from this cohort to minimize confounding factors.

### 2.2 Study data

Medical records were systematically reviewed to collect data on demographic characteristics, comorbidities (including diabetes mellitus, biliary disease, hepatitis and cirrhosis, history of intra-abdominal trauma or surgery, prior embolization and ablation treatments, and specific malignancy subtypes), clinical symptoms, imaging findings, laboratory parameters (such as alanine aminotransferase [ALT], aspartate aminotransferase [AST], albumin [ALB], total bilirubin [TBIL], alkaline phosphatase [ALP], and gamma-glutamyl transferase [GGT]), therapeutic interventions, drainage methods, abscess prognosis, discharge outcomes, and microbiological analysis results. Both the study groups and control groups were followed up for six months.

This study adhered to the principles outlined in the Declaration of Helsinki and was approved by the Institutional Ethical Committee (Ethics approval No. 2021298). Given the retrospective and anonymized nature of the data, the committee waived the requirement for written informed consent.

### 2.3 Microbiological culture and identification, antibiotic susceptibility

Pus and blood samples were cultured using the BACTEC™ FX (Becton Dickinson, Franklin Lakes, NJ). Bacterial identification and antibiotic susceptibility testing were conducted in accordance with the guidelines of the Clinical and Laboratory Standards Institute (CLSI). Microorganisms were identified via Matrix-Assisted Laser Desorption/Ionization-Time of Flight Mass Spectrometry (MALDI-TOF MS, Bruker Daltonics, Germany) and the PHOENIX-50 automated microbial identification and susceptibility system (Becton Dickinson, Franklin Lakes, NJ). Antibiotic susceptibility was assessed using the PHOENIX-50 system and confirmed by the Kirby-Bauer disk diffusion method on Müller-Hinton agar (Oxoid, Basingstoke, United Kingdom), with results reinterpreted in accordance with CLSI standards (2023). Quality control was ensured using reference strains including *Staphylococcus aureus* ATCC 25923, *Escherichia coli* ATCC 25922, and *Pseudomonas aeruginosa* ATCC 27853.

Bacterial identification was documented solely from the initial cultures of pus and blood obtained post-ablation. Common contaminants, such as *coagulase-negative staphylococci*, *Corynebacterium* sp*ecies*, *Bacillus* sp*ecies*, and *specific viridans group streptococci*, required repeated culturing from distinct samples to confirm their pathogenicity.

### 2.4 Statistical analysis

Statistical analyses were conducted using SPSS version 26.0 (IBM Corp., Armonk, NY, USA). Continuous variables were summarized as median and interquartile range, and comparisons between groups were performed using Student’s *t*-test or the Mann-Whitney *U* test, as appropriate. Categorical variables were analyzed using χ^2^ tests, Fisher’s exact tests, or continuity correction, depending on the data distribution. The identified variables were selected as candidates for univariate logistic regression analysis. Variables that exhibited statistical significance (*p* < 0.05) were subsequently incorporated into a multivariate logistic regression model using backward elimination to identify risk factors associated with liver abscess formation. Odds Ratios (OR) with 95% Confidence Intervals (CI) not encompassing 1, along with *p* < 0.05, were defined as indicating significant risk associations. Correlation coefficients (Point-Biserial or Phi) were selectively applied depending on the variable type. All statistical tests were two-tailed, and *P-*values < 0.05 were considered statistically significant.

## 3 Results

### 3.1 Baseline characteristics

The incidence of liver abscess following thermal ablation was 1.7%. **Table 1** summarizes the baseline characteristics of 61 patients with liver abscesses and their 61 propensity score-matched controls. Among the study group, 51 (83.6%) were male, with an age range of 29 to 79 years (mean age 58.0 ± 9.8 years). Eleven patients (18.0%) had metastatic liver cancer from various origins: colorectal (n=3), colon (n=2), bile duct (n=2), pancreatic (n=2), cervical (n=1), and gallbladder cancer (n=1); 18 patients (29.5%) had a history of biliary tract disease, specifically: 7 with stents, 5 with intrahepatic cholangiocarcinoma, 5 with bilioenteric anastomosis, and 1 with bile duct stones; 30 patients (49.2%) had undergone prior abdominal surgeries, including liver cancer resection (n=18), cholecystectomy (n=12), colorectal cancer resection (n=2), splenectomy (n=1), and liver transplantation (n=1). Compared to the control group, significant differences were observed in patients with liver abscesses regarding tumor type (HCC, *P* = 0.007; metastatic liver cancer, *P* = 0.047), history of diabetes mellitus (*P* = 0.035), biliary tract disease (*P* < 0.001), and history of abdominal surgery (*P* < 0.001). Laboratory findings revealed significantly higher levels of ALP (*P* = 0.001) and GGT (*P* = 0.008) in the liver abscess group compared to controls. No significant differences were found in ALT, AST, ALB, or TBIL levels between the two groups (**Table 1**).

**Table 1:** Baseline characteristics of patients with and without liver abscess.

Variables	Liver abscess n=61 (%)	Control group n=61 (%)	*P*-Value
Age, mean years ± SD	58.0 ± 9.8	58.0 ± 9.8	–
Sex, Male, n (%)	51 (83.6)	51 (83.6)	–
Tumor characteristics			
HCC, n (%)	45 (73.8)	57 (93.4)	0.007
ICC, n (%)	5 (8.2)	1 (1.6)	0.209
MLC, n (%)	11 (18.0)	3 (4.9)	0.047
Single/multiple tumors, n (%)	48/13	45/16	0.523
Located in the right/left lobe, n (%)	48/7	41/9	0.154
Liver function and complications			
Child-Pugh Class A/B, n (%)	40/20	43/18	0.138
Liver cirrhosis, n (%)	45 (73.8)	53 (86.9)	0.068
Biliary tract disease, n (%)	18 (29.5)	1 (1.6)	<0.001
Abdominal surgery, n (%)	30 (49.2)	11 (18.0)	<0.001
Diabetes mellitus, n (%)	26 (42.6)	15 (24.6)	0.035
Ever underwent TACE, n (%)	46 (75.4)	54 (88.5)	0.06
Multiple ablations, n (%)	51 (83.6)	49 (80.2)	0.638
Laboratory tests			
ALT, U/L (IQR)	27 (17.5, 38)	25 (17, 41)	0.682
AST, U/L (IQR)	32 (22, 50.5)	34 (26.2, 44)	0.728
ALP, U/L (IQR)	109 (78.5,168.5)	79 (68.5, 109.5)	0.001
GGT, U/L (IQR)	81 (47.5,168.5)	48 (29.5,102)	0.008
ALB(g/L), mean ± SD	37.5 ± 5.2	36.9 ± 4.9	0.712
TBIL, μmol/L (IQR)	17.8 (12.8,23.7)	17.3 (11.8,22.3)	0.575
Treatment and outcome			
Antibiotic prophylaxis, n (%)	4(6.6)	0	0.119
RFA/MWA ablation, n (%)	27/34	18/43	0.091
Outcome, n (%)	4(6.6)	0	0.119

### 3.2 Characteristics of patients with liver abscess

The median interval from thermal ablation to the diagnosis of liver abscess was 6 days (interquartile range, 3.5–20 days; range, 2–89 days). Enhanced CT confirmed liver abscesses in 52 patients (85.2%), characterized by necrotic liquefaction, gas accumulation, or air-fluid levels within the ablation area. All patients underwent percutaneous puncture drainage therapy; however, three patients required surgical drainage due to unsuccessful percutaneous attempts. Empirical antibiotic therapy typically involving latamoxef, third-generation cephalosporins (ceftriaxone and cefoperazone/sulbactam), or carbapenems (imipenem and meropenem), or metronidazole combinations, adjusted based on culture results. Follow-up evaluations via liver ultrasound or CT demonstrated resolution of clinical symptoms and signs, with abscess cavities disappearing or reducing to less than 2 cm in diameter prior to discharge. During the 6-month follow-up period, drainage catheters were successfully removed in 60 patients, while one patient experienced significant liver function decline due to prolonged catheter retention. Abscess resolution occurred in 56 (91.8%) within 7–60 days, with one recurrence and four deaths during the follow-up period. (**Table 2**).

**Table 2:** Characteristics of patients with liver abscess formation after ablation.

Variables	Liver abscess n=61 (%)
Diagnosis time	6 (3.5, 20)
Clinical manifestations	
Fever	53 (86.9)
Chills	23 (37.7)
Abdominal distension/pain	29 (47.5)
Fatigue	8 (13.1)
Nausea/vomiting	5 (8.2)
Tachycardia	4 (6.6)
Altered consciousness	2 (3.3)
CT findings	
Gas-formation	49 (80.3)
Air–fluid levels	8 (13.1)

### 3.3 Analysis of risk factors associated with the formation of post-ablation liver abscesses

Univariate analysis revealed diabetes mellitus, history of abdominal surgery, biliary tract disease, HCC with metastasis, elevated ALP and GGT levels as significantly associated with the development of liver abscesses following thermal ablation (all *P*<0.05). Meanwhile, correlation coefficient analysis revealed that biliary tract diseases were most strongly associated with the occurrence of liver abscess (r = 0.42). In contrast, diabetes and abdominal surgeries exhibited weaker associations with liver abscess formation (r = 0.18 and r = 0.25, respectively).

Multivariate logistic regression analysis revealed that diabetes mellitus (OR 3.215; 95% CI 1.330–7.771; *P*=0.009), a history of abdominal surgery (OR 2.810; 95% CI 1.074–7.353; *P* = 0.035), biliary tract disease (OR 18.832; 95% CI 2.291–154.795; *P* = 0.006), and elevated ALP levels (OR 1.010; 95% CI 1.002–1.019; *P* = 0.014) are independent risk factors for post-ablation liver abscess formation. The association between HCC with metastasis and higher GGT levels did not retain statistical significance in the multivariate analysis (**Table 3**).

**Table 3:** Risk factors associated with post-ablation liver abscess.

Variables	Univariate analysis			Multivariate analysis	
	OR (95%CI)	*r*-value	*p-*value	OR (95%CI)	*P-*value
Diabetes mellitus	2.278 (1.052-4.934)	0.18	0.037	3.215 (1.330-7.771)	0.009
Abdominal surgery	2.877 (1.222-6.771)	0.25	0.016	2.810 (1.074-7.353)	0.035
Biliary tract disease	25.116 (3.229-195.374)	0.42	0.002	18.832 (2.291-154.795)	0.006
ALP	1.011 (1.004-1.019)	0.35	0.003	1.010 (1.002-1.019)	0.014
HCC	0.215 (0.067-0.693)	-0.27	0.01	–	–
MLC	4.52 (1.123-16.106)	0.21	0.033	–	–
GGT	1.005 (1.001-1.009)	0.28	0.028	–	–

### 3.4 Microbiology

Among the 61 patients, monomicrobial cultures were observed in 46 patients (75.4%), polymicrobial cultures with two organisms in 13 (21.3%), and three organisms in 2 (3.3%). Consequently, a total of 78 isolates were identified from the pus cultures. The distribution of isolates indicated that Gram-negative bacteria predominated (75.6%), with *Escherichia coli* (30.8%) and *Klebsiella pneumoniae* (20.5%) being the most common. Gram-positive bacteria accounted for 24.4% of isolates, primarily *Enterococcus faecium* (12.8%) and *Enterococcus faecalis* (3.8%). Mixed bacterial infections were identified in 15 cases of liver abscesses, including combinations of both Gram-negative and Gram-positive bacteria (6 cases).

Positive bacterial cultures were identified in both blood and pus samples from 21 patients. Single organisms were isolated in 18 cases, while multiple organisms were found in the remaining 3. *Escherichia coli* was the most common isolate from blood cultures, accounting for 41.7% (10/24 isolates), followed by *Enterococcus faecium* at 16.7% (4/24) and *Klebsiella pneumoniae* at 12.5% (3/24) (**Table 4**).

**Table 4:** Organisms identified from liver abscess pus and blood cultures in patients undergoing ablation therapy.

Bacteria	Pus culture n=78 (%)	Blood culture n=24 (%)
*Gram-negative bacteria*	59 (75.6%)	17 (70.8%)
*Escherichia coli*	24 (30.8%)	10 (41.7%)
*Klebsiella pneumoniae*	16 (20.5%)	3 (12.5%)
*Pseudomonas aeruginosa*	4 (5.1%)	0
*Proteus* spp.	3 (3.8%)	1 (4.2%)
*Acinetobacter* spp.	2 (2.6%)	0
*Klebsiella oxytoca*	2 (2.6%)	1 (4.2%)
*Citrobacter freundii*	2 (2.6%)	0
Others*	6 (7.6%)	2 (8.4%)
*Gram-positive bacteria*	19 (24.4%)	7 (29.2%)
*Enterococcus faecium*	10 (12.8%)	4 (16.7%)
*Enterococcus faecalis*	3 (3.8%)	2 (8.3%)
*Staphylococcus aureus*	2 (2.6%)	1 (4.2%)
Others*	3 (3.9%)	0

Others* (Gram-negative): Serratia marcescens, Salmonella enteritidis, Enterobacter asburiae, Achromobacter xylosoxidans, Enterobacter cloacae, and Morganella morganii. Others (Gram-positive): Streptococcus spp. and Staphylococcus epidermidis.

Among the 15 liver abscesses with mixed infections, Gram-negative and Gram-positive bacteria coexisted in 6 cases. Concordant blood and pus cultures were observed in 4 patients; 3 had identical species distributions, while one case isolated *Escherichia coli* and *Enterococcus faecalis* from pus but only *Escherichia coli* was isolated from blood culture (**Table 5**).

**Table 5:** Distribution of mixed bacterial species in liver abscess pus and blood cultures from patients undergoing ablation therapy.

Bacterial combination	Pus culture (n=15)	Blood culture (n=3)
Gram-negative combinations		
*Escherichia coli+Klebsiella pneumoniae*	2	2
*Klebsiella pneumoniae+Pseudomonas aeruginosa*	2	0
*Klebsiella pneumoniae+klebsiella oxytoca*	1	0
*Escherichia coli+Proteus vulgaris*	1	0
*Klebsiella pneumoniae+Proteus vulgaris + morganella morganii*	1	0
Mixed gram-negative and gram-positive		
*Escherichia coli+Enterococcus* spp.	3	0
*Escherichia coli+Staphylococcus aureus*	1	1
*Klebsiella pneumoniae+Enterococcus faecium*	1	0
*Citrobacter freundii+Streptococcus anginosus*	1	0
*Acinetobacter junii+Enterococcus faecium*	1	0
*Klebsiella pneumoniae + Escherichia coli + Enterococcus faecium*	1	0

### 3.5 Antibiotic susceptibility analysis

From the pus and blood samples of 61 patients with liver abscesses, a total of 34 strains of *Escherichia coli* were isolated. Antimicrobial susceptibility testing demonstrated high sensitivity to commonly prescribed antibiotics such as Amikacin, Cefoxitin, Ceftazidime, Imipenem, Meropenem, and Piperacillin-tazobactam. Notably, 14 strains (41.2%) were identified as extended-spectrum beta-lactamase (ESBL) producers. One strain (2.9%) exhibited resistance to Carbapenems, while all strains remained susceptible to Amikacin.

**Figure 1:**
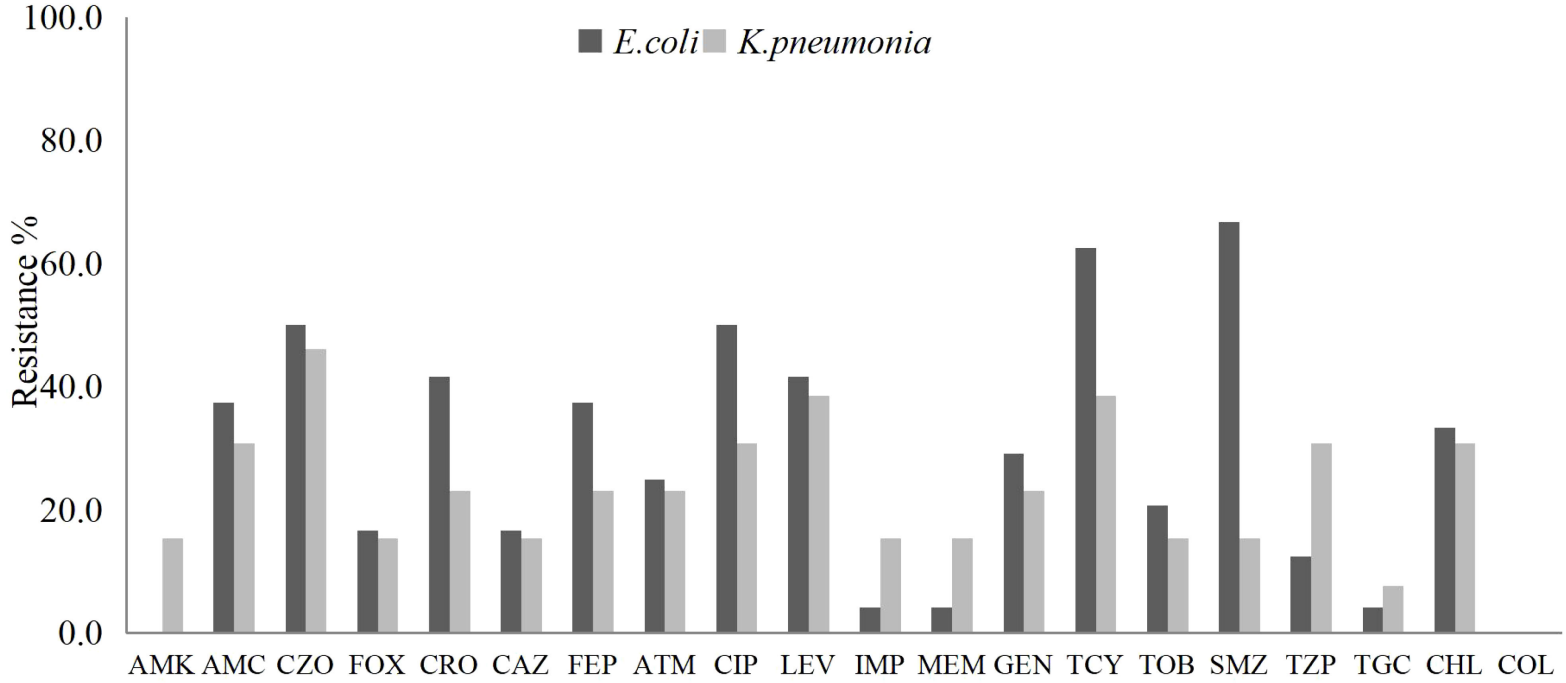
The antibiotic resistance rates of *E. coli* and *K. pneumoniae* isolated from liver abscess pus and blood samples against commonly used clinical antibiotics.

Nineteen strains of *Klebsiella pneumoniae* were isolated, and demonstrated high susceptibility (over 80%) to Amikacin, Cefoxitin, Ceftazidime, Imipenem, and Meropenem. The susceptibility rate to Piperacillin-tazobactam was 78.9%. Notably, four strains (21.1%) were identified as ESBL producers, while three strains (15.8%) exhibited resistance to Carbapenems (**Figure 1**).

**Figure 2:**
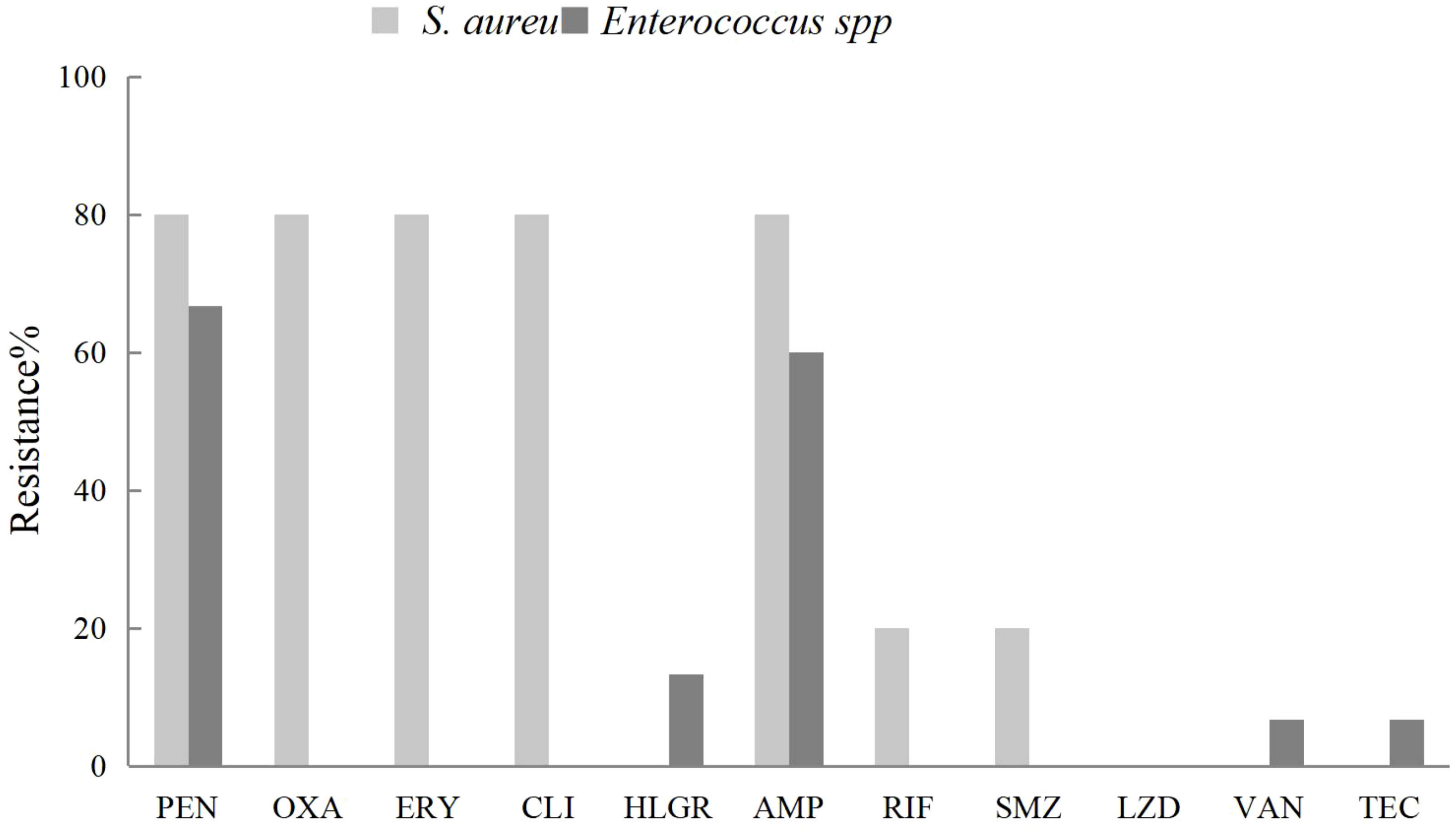
The antibiotic resistance rates of *Enterococcus feacium* and *Enterococcus farcalis* isolated from liver abscess pus and blood against commonly used clinical antibiotics.

Fourteen strains of *Enterococcus faecium* were isolated from pus and blood samples of liver abscess patients. These strains exhibited high susceptibility to high-dose gentamicin, linezolid, vancomycin, and teicoplanin. Notably, one strain (7.1%) demonstrated resistance to vancomycin. Five strains of *Enterococcus faecalis* were isolated, all showing complete susceptibility to linezolid, vancomycin, and teicoplanin. Compared with *Enterococcus faecium*, *Enterococcus faecalis* exhibited greater susceptibility to penicillin, ampicillin, and ciprofloxacin (**Figure 2**).

## 4 Discussion

As thermal ablation becomes increasingly important for treating primary and metastatic liver cancer, managing its associated complications has become a clinical priority (Liang et al., 2009; Spiliotis et al., 2021). Although liver abscess is a relatively common complication following thermal ablation, its low incidence rate has resulted in very limited reports of positive cases in the existing literature. This study is one of the few in this field to include all cases with definitive bacterial culture results, thereby providing a crucial reference for further investigating the characteristics and pathogenesis of liver abscesses.

In this study, the majority of patients who developed post-ablation liver abscesses were male and aged 60 years or older. The predominant symptoms included persistent fever, chills, and abdominal pain or distension. Nevertheless, distinguishing these symptoms from the common post-ablation clinical manifestations such as fever and abdominal pain, can be particularly challenging in the early stages (Park et al., 2017). Moreover, the presence of gas and necrotic lesions after TAE/TACE and ablation therapy further complicates the immediate differentiation between liver abscesses and tumor necrosis syndrome using imaging modalities (Mezhir et al., 2011; Jia et al., 2018). Consequently, early diagnosis of post-ablation liver abscesses poses a significant challenge. In this study, the average time to diagnosis was 6 days, with the longest recorded diagnosis time extending up to 3 months. Understanding the risk factors associated with the formation of these abscesses can facilitate the implementation of preventive measures and expedite diagnosis, ultimately leading to improved patient outcomes. Previous studies on the risk factors of post-ablation liver abscesses have reported inconsistent findings, likely attributable to variations in study populations (Choi et al., 2005; Su et al., 2016; Wang et al., 2024). In this study, our results indicate that diabetes mellitus, a history of abdominal surgery, biliary disease, and elevated ALP levels are independent risk factors for developing post-ablation liver abscesses.

It is well established that diabetic patients are particularly vulnerable to liver abscesses, likely due to their compromised immune status (Tian et al., 2012; Li et al., 2018; Guo et al., 2023). previous studies analyzed complications in 1,136 patients following MWA and reported that 40% of patients with liver abscesses had underlying diabetes mellitus (Liang et al., 2009). Other studies (Su et al., 2016) observed 423 patients who underwent thermal ablation and reported that among the 12 patients who developed liver abscesses, 5 (41.7%) had a history of diabetes. Our study aligns with these findings: 42.6% of patients who developed liver abscesses post-ablation had diabetes, compared to 24.6% in the control group. However, the results of this study indicate that there is only a weak correlation between diabetes and the formation of liver abscesses(r=0.18). Therefore, further research is needed on the link between glycemic control and liver abscess occurrence. Nonetheless, strict blood sugar management remains crucial for diabetic patients undergoing ablation therapy, both pre- and post-operation.

Many liver cancer patients have a history of prolonged liver disease and frequently undergo various surgical treatments prior to ablation therapy. In this study, 54.1% of patients who developed post-ablation liver abscesses had previous abdominal surgeries such as liver resection, cholecystectomy, splenectomy, and colorectal cancer resection. These surgeries may increase the risk of liver abscesses, potentially due to its effects on immune function and alterations in gut microbiota, which can facilitate bacterial entry into the liver via multiple pathways (Lai et al., 2015; Tsai et al., 2016; Zhang et al., 2018). However, the statistical analysis results of this study reveal only a weak correlation between prior surgical history and the development of liver abscess (r=0.25), Thus, further large-scale studies are needed to confirm if surgical history is an independent risk factor.

Biliary disease has long been recognized as a leading cause of liver abscesses, accounting for 50-60% of cases that originate from the biliary system (Lai et al., 2013; Song et al., 2020). This association primarily arises from bacterial contamination due to biliary diseases and bile stasis caused by malignant biliary strictures or anastomotic strictures (Zhang et al., 2015). Lin’s study further confirms that biliary diseases represent an independent risk factor for post-ablation liver abscess, a finding that aligns closely with our research results (Lin et al., 2024). Additionally, some researchers suggested that RFA should be cautiously considered in patients who have undergone choledochojejunostomy, particularly when tumor nodules are not peripheral and preoperative ALP levels are elevated (Iida et al., 2014). Therefore, prior to performing thermal ablation in patients with biliary diseases, a thorough evaluation and optimization of their clinical status are essential to minimize the risk of complications.

ALP is an important biomarker for evaluating liver injury. Elevated ALP levels often indicate pathological changes in the liver or biliary system, potentially due to bile outflow obstruction, which may cause intestinal bacteria to colonize the liver. The results of this study are consistent with Kwak et al., who reported a significant link between elevated serum ALP and post-ablation liver abscesses (Kwak et al., 2023). However, with only 4 out of 253 patients developing liver abscess, the low incidence limited the statistical power. Therefore, the correlation between ALP levels and the occurrence of post-ablation liver abscesses remains to be further validated through studies with larger sample sizes and more rigorous designs.

For patients with confirmed liver abscesses, the standard of care entails percutaneous drainage combined with appropriate antibiotic therapy (Mezhir et al., 2010). However, antibiotic susceptibility testing generally requires 3 to 5 days or longer. During this period, while awaiting microbiological results, initiating empiric broad-spectrum antibiotic therapy is critical for patient outcomes (Sutcliffe et al., 2015). Understanding the pathogens and their resistance profiles associated with liver abscesses helps guide rational antibiotic selection and improve patient outcomes.

Recent data indicate that Gram-negative bacteria, particularly Enterobacteriaceae such as *Escherichia coli* and *Klebsiella pneumoniae*, have emerged as the predominant pathogens responsible for post-ablation liver abscesses (Su et al., 2016; Wang et al., 2024). This study corroborates these findings, further revealing ESBL positivity rates of 41.2% for *Escherichia coli* and 21.1% for *Klebsiella pneumoniae*. Additionally, these bacteria showed low sensitivity to quinolones, suggesting that cephalosporins and quinolones may no longer be suitable for empirical treatment. Carbapenem antibiotics are widely regarded as the most effective treatment for Enterobacteriaceae infections (Ding et al., 2023). In this study, *Escherichia coli* and *Klebsiella pneumoniae* also exhibited high susceptibility to carbapenems. However, evidence shows that increased carbapenem use has significantly reduced susceptibility in Enterobacteriaceae, especially Klebsiella pneumoniae (Hu et al., 2024). Meanwhile, this study demonstrated that Gram-negative bacteria exhibited relatively lower resistance rates to Amikacin, Cefoperazone, Ceftazidime, and Piperacillin/Tazobactam, indicating that these antibiotics may serve as potential alternative options in clinical practice.

Given that 24.4% of the isolates *were* Gram-positive bacteria and 24.6% of cases involved Polymicrobial infections (including 9.8% with mixed Gram-negative and Gram-positive infections), clinicians should consider broad-spectrum antibiotics effective against both Gram-negative and Gram-positive organisms when initiating empirical or prophylactic therapy. Moreover, ongoing microbiological surveillance combined with culture-based susceptibility testing continues to be critical for guiding the judicious use of antimicrobial agents.

Due to the limited sample size, this study did not explore the potential relationship between identified risk factors and microorganisms isolated from liver abscesses. Previous research shows diabetic patients are at higher risk for *Klebsiella pneumoniae*-induced liver abscesses (Guo et al., 2023). Biliary tract disease, a key risk factor, disrupts bile flow and promotes bacterial translocation. Studies also indicate biliary-origin pyogenic liver abscesses are mainly associated with *Escherichia coli* (Shi et al., 2018). These findings highlight the prevalence of enteric pathogens like Escherichia coli and Klebsiella pneumoniae in our cultures.

Our study had some limitations. Firstly, we focused exclusively on patients with positive pus cultures, excluding those with negative cultures or only positive blood cultures, which may have affected the generalizability of our findings and incidence estimation of post-ablation liver abscesses. Secondly, our analysis did not consider confounding factors like age and gender, which could influence outcomes. Thirdly, as a single-center retrospective study, it is prone to selection and information biases. Lastly, the relatively small sample size and short follow-up period may have introduced additional limitations into our findings. Subsequently, more in-depth research will be carried out on the above-mentioned deficiencies, and systematic analysis and elaboration will be conducted.

## 5 Conclusion

History of diabetes, prior abdominal surgery, biliary tract diseases, and elevated ALP levels are key risk factors for liver abscess after ablation therapy. Clinicians should assess high-risk patients preoperatively, optimize their condition, and tailor treatment plans. The main bacteria causing post-ablation liver abscesses are Gram-negative, such as *E. coli* and *K. pneumoniae*. Clinically, strengthen specimen culture collection and antimicrobial resistance monitoring, and choose appropriate antibiotics based on sensitivity test results to control infection and prevent drug resistance.

## Data Availability

The original contributions presented in the study are included in the article/supplementary material. Further inquiries can be directed to the corresponding author.
